# Straightening the crooked: intraspecific divergence of stem posture control and associated trade-offs in a model conifer

**DOI:** 10.1093/jxb/erab535

**Published:** 2021-12-05

**Authors:** Rosario Sierra-de-Grado, Valentin Pando, Jordi Voltas, Rafael Zas, Juan Majada, Jose Climent

**Affiliations:** 1 ETSIA, Universidad de Valladolid, Avda de Madrid 44, 34004 Palencia, Spain; 2 iuFOR, University Institute for Research in Sustainable Forest Management, Avda de Madrid 44, 34004 Palencia, Spain; 3 Department of Crop and Forest Sciences, University of Lleida, Av. Alcalde Rovira Roure 191, E-25198 Lleida, Spain; 4 Joint Research Unit CTFC–AGROTECNIO–CERCA, Av. Alcalde Rovira Roure 191, E-25198 Lleida, Spain; 5 Misión Biológica de Galicia (MBG-CSIC), Apdo 28, 36143 Pontevedra, Spain; 6 CETEMAS, Pumarabule s/n, Carbayín, 33936 Asturias, Spain; 7 Centro de Investigaciones Forestales (INIA-CSIC), Ctra. A Coruña km 7.5, 28040 Madrid, Spain; 8 University of Cambridge, UK

**Keywords:** Adaptation, biomechanics, integrated phenotype, intraspecific variation, life history, provenances, stem straightness, trade-offs

## Abstract

Although the straightening capacity of the stem is key for light capture and mechanical stability in forest trees, little is known about its adaptive implications. Assuming that stem straightening is costly, trade-offs are expected with competing processes such as growth, maintenance, and defence. We established a manipulative experiment in a common garden of *Pinus pinaster* including provenances typically showing either straight-stemmed or crooked-stemmed phenotypes. We imposed a bending up to 35º on plants aged 9 years of both provenance groups and followed the straightening kinetics and shoot elongation after releasing. Eight months later, we destructively assessed biomass partitioning, reaction wood, wood microdensity, xylem reserve carbohydrates, and phloem secondary metabolites. The experimental bending and release caused significant, complex changes with a marked difference between straight- and crooked-type plants. The straight-type recovered verticality faster and to a higher degree and developed more compression wood, while displaying a transitory delay in shoot elongation, reducing resource allocation to defence and maintaining the levels of non-structural carbohydrates compared with the crooked type. This combination of responses indicates the existence of intraspecific divergence in the reaction to mechanical stresses that may be related to different adaptive phenotypic plasticity.

## Teaser

Bending triggered contrasting responses between straight- and crooked-stemmed provenances of maritime pine linked to the straightening process, showing different trade-offs with other features related to growth, maintenance, and defence.

## Introduction

Forest trees are long-lived, extremely slender sessile living beings that must cope with highly variable mechanical stresses like wind, rainfall, snow, and ice as well as support their own continuously increasing weight. To ensure long-term survival, trees must have both mechanical resistance (structural function) and mechanisms to actively reorient themselves (motor function) to regain a stable position (Martone *et al.*, 2010; Fournier *et al.*, 2013).

While stem straightness is a key trait affecting the economic value of wood production, we still know very little about the adaptive implications of growing straight under different environmental conditions. In boreal conifers, ecotypes from higher latitudes or altitudes are more slender and have straighter stems than those from lower latitudes or altitudes, and these differences have long been interpreted as adaptations to snow and wind (Timell, 1986; González-Martínez *et al.*, 2004). Comparative studies between tropical tree species showed that straight stems and dense woods were associated with greater longevity and late reproduction, while species with crooked stems showed the opposite traits (Van Gelder *et al.*, 2006; Poorter *et al.*, 2010). The causes of the great variability of forms in some species are, however, far from completely understood (e.g. *Picea abies*; Geburek *et al.*, 2008). In particular, in temperate Mediterranean species, the adaptive role of stem straightness (and of tree shape in general) remains mostly unexplored (Barthélémy and Caraglio, 2007). A study in *Pinus pinaster* Ait. found that selection by tree form (including straight stems, high apical dominance, and thin branches) had a correlated response of delayed reproduction (Santos-del-Blanco *et al.*, 2015). In agreement with the postulates of life-history theory, a general link between straight stems and larger size at maturity, greater longevity, and late reproduction is, therefore, plausible (Stearns, 1976). Moreover, from a physiological point of view, life-history strategies favouring straight stems in long-lived, tall trees imply higher relative allocation to long-term structural components (the main stem and the root system) and, hence, reduced allocation to branches and photosynthetic biomass (Schwilk and Ackerly, 2001; Tapias *et al.*, 2004; Pausas *et al.*, 2004). This strategy would improve tree stability and tolerance to mechanical disturbances such as those caused by snowstorms or wind, since these traits can integrate at the tree scale by combining tree size and shape together with wood properties, as described by Fournier *et al.* (2013). Therefore, we cannot discount the relevance of snow load as a key selective agent favouring straight growth habit and high allocation to stems in continental and mountain Mediterranean regions, which would explain, at least in part, the large intraspecific variation in stem form found in many Mediterranean species (Climent and Sierra-de-Grado, 2017).

Plants detect their relative position in the gravitational field through very sensitive gravitropic sensing mechanisms. When a stem is tilted, gravitropic sensing is mediated by statoliths that interact with the cell membrane of statocytes, triggering a signal transduction in which Ca^2+^ and other second messengers are involved, in turn leading to an asymmetric efflux of auxin between the upper and lower sides of the tilted stem, causing a differential elongation of the cells that finally leads the stem to curve upward (Moulia and Fournier, 2009; Coutand, 2010; Fournier *et al.*, 2013; Schüler *et al.*, 2015; Bastien *et al.*, 2015; Moulia *et al.*, 2019). This rapid reaction is driven by primary growth, but in woody plants, a slower reaction driven by secondary growth also takes place, involving asymmetric radial growth, the formation of reaction wood, and a resulting asymmetry of maturation strains and growth stresses between opposite sides of the stem section (Fournier *et al.*, 1994, Gardiner *et al.*, 2014). This asymmetry of stresses produces an active change in the stem curvature (gravitropic response) and, in turn, provokes a countercurvature (autotropic response) (Fournier *et al.*, 1994; Sierra-de-Grado *et al.*, 2008; Moulia and Fournier, 2009; Ba *et al.*, 2010). These processes allow trees to regulate their postural control, a key factor for their mechanical stability that is directly involved in stem straightness. As a result, reaction wood formation in woody plants, i.e. tension wood in angiosperms and compression wood in gymnosperms, is of paramount importance in the control of posture (Gardiner *et al.*, 2014).

Therefore, the straightness of a tree trunk depends not only on external factors inducing curvature, but also on intrinsic factors, probably genetically controlled, governing resource allocation and tree architecture and linked to the ability of the tree to resist mechanical stresses and eventually recover a stable, straight position. While stem form in forest trees has been widely reported to be under moderate genetic control (i.e. moderate heritability and responding to artificial selection; see, for example, White *et al.* (2007) and references therein), there is experimental evidence supporting that ecotype differentiation and heritability of stem straightness increase substantially in manipulative experiments, including the application of a mechanical stress that triggers the re-straightening process (e.g. in *P. pinaster*; Sierra-de-Grado *et al.*, 1997).

These morphological and physiological changes in response to mechanical stimuli can be studied under the paradigm of adaptive plasticity, i.e. the extent of tree phenotypic responses increasing fitness when facing environmental change (Chambel *et al.*, 2005; Moulia *et al.*, 2006; Nicotra *et al.*, 2010; Fournier *et al.*, 2013). The structural reinforcement in response to mechanical stresses can vary in different ecological contexts at the expense of other functions (Read and Stokes, 2006). From an evolutionary perspective, it is therefore relevant to study whether the functions of mechanical support and re-straightening of the trunk trade off with other basic functions such as growth, reproduction, and defences against biotic agents, hence implying differential resource allocation patterns. Unveiling the covariation structure between stem straightness and other life-history traits will help in understanding their cost–benefit balance and the adaptive relevance of straight stems and re-straightening processes (Worley *et al.*, 2003; Roff and Fairbairn, 2007).

During the past decades, maritime pine (*P. pinaster* Ait.) has become the main Mediterranean model conifer for eco-genetic studies (González-Martínez *et al.*, 2004; Alía *et al.*, 2014; Serra-Varela *et al.*, 2015). This species has a highly fragmented distribution between SW Europe and NW Africa, covering a wide range of environments and combining high populational differentiation with high intra-population diversity (González-Martínez *et al.*, 2004). Importantly, this species displays a particularly high variability of stem forms both between and within populations (Alía *et al.*, 1995; Sierra de Grado *et al.*, 1999). Population differentiation in stem form and branching habit is evident in common gardens established in France, Spain, Portugal (see for example Climent *et al.* (2021) and references therein), and also in SW Australia (Butcher and Hopkins, 1993). Little is known, however, about the contribution of different mechanistic processes (e.g. allocation priorities to mechanical support, ability to recover verticality after disturbance, etc.) to the inter-population variation in stem straightness, and whether different stem form habits may be integrated in phenotypic syndromes affecting other life-history traits. Manipulative experiments forcing tree curvatures in common gardens may help to unveil the differential behaviour of straight and crooked populations when faced with mechanical stimuli.

The aim of this work is to understand the extent of plastic responses and trade-offs among traits involved in mechanical stability (recovery of verticality), growth, and defence functions in *P. pinaster.* To attain this objective, we performed an artificial bending experiment in a young provenance trial that included a selected group of provenances with extreme form differences (straight versus crooked), which were chosen based on previous results from more representative, older common gardens (Alía *et al.*, 1995, 1997). Our main hypotheses were, first, that stem straightening is a costly process that must modify biomass allocation and trade off with growth and investment in defensive secondary metabolites, and second, that straight provenances should show higher allocation to the main stem, higher plastic recovery after mechanical disturbance, and a more efficient usage of stored resources (i.e. less consumption) related to the straightening process compared with crooked provenances.

## Materials and methods

The experiment was conducted in a common garden of 9-year-old *P. pinaster* provenances located in La Cistérniga, central Spain (41° 35ʹ 38.6 N 4° 39ʹ 48.9 W), characterized by a continental Mediterranean climate and clay soil. The original common garden experimental design consisted of completely randomized blocks, but the high mortality during previous years made the design so unbalanced that it led us to disregard the block structure for statistical purposes. Seven provenances were chosen, four among those typically showing straight-stemmed phenotypes (Burgos-Soria, Gredos, Leiria, Tamjout; from now on referred to as straight-type) and three among the crooked-stemmed phenotypes (Oña, Meseta Castellana, Almijara; from now on referred to as crooked-type) following the ranking of stem straightness evaluated in five provenance trials in Spain (Alía *et al.*, 1995). Total number of the trees in the experiment was 59, with a range between 5 and 12 trees per provenance (Supplementary Table S1).

**Fig. 1.**
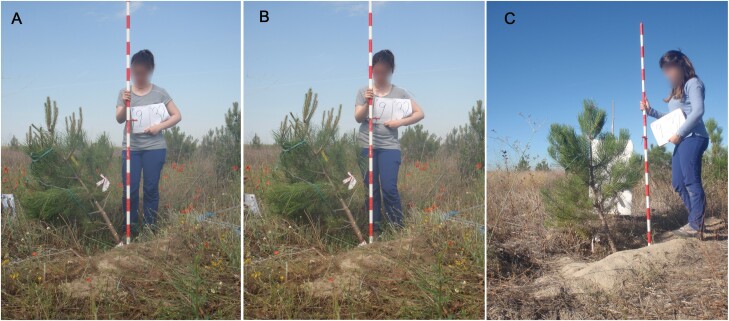
Examples of images taken on a bent plant just before (A) and after (B) releasing, and at the end of the experiment (C).

We established a manipulative experiment consisting in an artificial temporal bending of half of the trees (randomly chosen) of each population, followed by releasing. The remaining half of the trees were left untreated and acted as controls. Plants were bent southwards to an inclination of 35º with respect to the vertical using a rope attached under the fourth branch whorl (Fig. 1). We chose this angle to generate a maximum response in the plants, based on the studies by Yamashita *et al.* (2007) in *Cryptomeria japonica* and Herrera *et al.* (2010) in *P. pinaster*. Control and bent plants were chosen considering a similar range of plant size in plants pertaining to the two provenance groups (30 bent and 29 control, with 14–15 plants for each provenance group). The range of tree sizes was large due to an important micro-environmental variation within the trial (basal diameters varying between 1 and 10 cm and heights between 0.5 and 2.5 m). The bending was induced at the end of April 2017 (i.e. at the start of the growing season) and plants were released at the beginning of June of the same year. The straightening kinetics and height growth were followed after releasing (Supplementary Fig. S1A). All plants were harvested in January of the following year for the analysis of the following traits: amount of reaction wood, wood microdensity, biomass allocation, non-structural carbohydrates (soluble sugars and starch), and secondary defensive metabolites (total polyphenolics and condensed tannins in the phloem). When harvested, the cardinal directions were marked in the trunks.

### Elongation and straightening kinetics

**Fig. 2.**
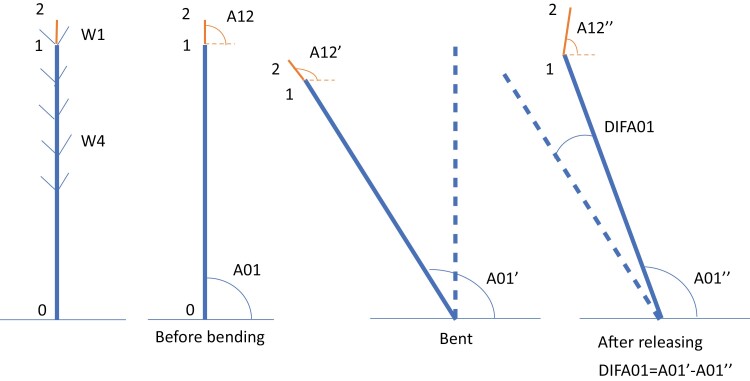
Schematic illustration of the measured traits used to follow the recovery process after bending. In each plant three key points were identified in the stem: point 0 at the basal point of the stem; point 1 at the insertion of the first whorl of branches; and point 2 at the apex. These three points delimited two different stem segments: segment 01 (stem developed before the year of the experiment), and segment 12 (portion of the stem elongated during the course of the experiment). The angles with respect to the horizontal of these two segments (A01 and A12) were used to follow the straightening process of each tree. DIFA01 and DIFA12 are the difference of A01 and A12 at each date with respect to A01 and A12 just after releasing (DIFA01=A01ʹ−A01″; DIFA12=A12ʹ−A12″).

We buried a metal tube at the east of each plant to support a camera and take images periodically. In each photograph, we included a metric scale located vertically at the base of each tree with a bubble level. We marked three key points in the stems to track the recovery process in the images: point 0 at the base of the stem; point 1 at the base of the first whorl of branches; and point 2 at the apex. For analysis purposes, we considered two parts of the stems (Fig. 2): the basal part, which developed during the previous years of plant life (corresponding to the segment between points 0 and 1), and the apical part, which elongated during the experiment (corresponding to the segment between points 1 and 2). Starting the day the treatment was applied (day (D) 0), images were taken every 7 d during the first month (D7, D14, D21, D28, and D35), and at increasing time intervals thereafter (D45, D56, D80, D101, D122, and D143). At each measuring date (*i* d after releasing), we obtained the length of the apical segment (LENG_i_). The movement of the stem along the experiment was assessed by the angles (deviations from verticality) of the basal and apical parts (A01 and A12; Fig. 2). Angle A01 indicates the verticality of the trunk and A12 that of the stem in the apical elongating segment. We also computed the differences of angles in each date with respect to the angles just after releasing (DIFA01 and DIFA12). Metric and angle measurements were adjusted with the scales in each image. All measurements were performed using Photoshop 5.5 and 6.0 (Adobe Systems Inc., 2000).

### Biomass partitioning

In January (143 d after bending), all trees were harvested by cutting the stems at the ground level. We did not extract the rooting system, since our field test conditions made it infeasible in a practical and/or reliable way. The aerial part of the trees was then dried at 65 °C to constant mass (around 8 d) to estimate biomass allocation to the main stem, leaves, and branches. The respective relative mass fractions (S_1_MF, LMF, and S_2_MF) with respect to total biomass (TDW) were then calculated. The leader shoot mass fraction (S1_L_MF) was also calculated.

### Compression wood and diameter growth

Three transverse 20 mm-thick sections were obtained at the basal (ground level, i.e. at point 0, Fig. 2), middle (under the 4-year-old branch whorl, i.e. around the middle point of segment 01) and upper (under the 1-year old branch whorl, i.e. at point 1) parts of each trunk. The sections were sanded with abrasive paper until tree rings were clearly visible and scanned at 800 × 800 pixels resolution. The compression wood area was identified by darker colour under a WF10X/22 Wide Field Stereo Microscope and transferred to the scanned images. The CW area and the total area of the ring formed in the year of the experiment were measured on the scanned images using ImageJ (Schneider *et al.*, 2012) to estimate the percentage of compression wood area over the total yearly ring area (pCW).

We also measured the xylem diameter and the diameter of xylem corresponding to the year before the experiment to estimate the diameter growth during the year of the experiment.

### Wood microdensity

An extra 20 mm-thick slice from the base of the trunk was sampled and left in a chamber at 20 ºC and 65% humidity for a week. Once conditioned, a 1 mm-thick strip following the north–south orientation was obtained using a precision saw and used for X-ray analysis. Fifty-five trees were analysed (four trees were discarded due to their small size). Resin was previously extracted from the samples with ethanol continuously boiling under reflux for 12 h and then dried. The samples were then scanned with Itrax Multiscaner X-ray equipment (Itrax, Cox Analytics, Sweden). The radiographic images obtained were analysed using the WinDendro program (Regent Instruments, Canada). Wood density (WDENS) was obtained for each tree at the north and south extremes of the trunk in the wood growth ring corresponding to the year of the experiment.

### Non-structural carbohydrates (soluble sugars and starch)

An additional 20 mm-thick cross-section of the trunk below the fourth whorl of branches was separated at harvest, kept cold until reaching the laboratory, and then placed in a microwave at 600 W for 90 s to denature the enzymes (Hoch *et al.*, 2002). Then, the sections were dried in the oven at 65°C to constant mass, debarked and ground in a ball mill to obtain sawdust for the determination of non-structural carbohydrates. Four analytical repetitions were done per section, two corresponding to its north side and two corresponding to its south side. Soluble sugars were extracted from about 50 mg of sample with 80% ethanol in a shaking water bath at 60 °C. The concentration of soluble sugars in the supernatant obtained after centrifugation (SUGAR, %) was determined colourimetrically at 490 nm in a Bio-Rad model 680 microplate reader, using the phenol–sulphuric acid method described by Buysse and Merckx (1993). After ethanol extraction, the remaining undissolved precipitate was digested with amyloglucosidase to reduce starch to glucose, as described by Palacio *et al.* (2007). The resulting glucose was measured by the method of Buysse and Merckx (1993), from which the original starch concentration was obtained (STARCH, %).

### Secondary defensive metabolites

Just before harvesting the plants, we took a rectangular phloem sample of around 2 cm^2^ with a knife, under the fourth branch whorl at the south side of the stem. The sample was immediately put in an Eppendorf tube and stored in liquid nitrogen until analysis in the laboratory. The concentration of total polyphenolics and condensed tannins in these phloem samples was determined as in Moreira *et al.* (2009) with slight modifications. Briefly, 20 mg of finely ground freeze-dried sample was extracted with 1 ml of aqueous methanol (1:1, v:v, HPLC grade, HiperSolv Chromanorm) in 1.1 ml reaction tubes (VWR, Microtiler cat. no. T100-25). The tubes were vortexed, sonicated for 15 min, centrifuged at 2013 *g* for 20 min (Eppendorf Centrifuge 5804, Germany) and the supernatant saved. A diluted aliquot was allowed to react with Folin reagent (cat. no. 1.09001.0500, Merck, Germany) and sodium carbonate (cat. no. 131648.1210, Panreac, Germany) for 2.5 h. The absorbance was then measured at 740 nm in a microplate reader (Bio-Rad model 680). Concentration of total phenolics (PHEN, mg g^−1^) was estimated using tannic acid (Panreac cat. no. 141065) as standard and expressed as tannic acid equivalents. Condensed tannins were analysed in the same methanolic extract following Sampedro *et al.* (2011). The methanolic extract was mixed with butanol (VWR AnalR Normapur, cat. no. 20810.323) and ferric ammonium sulphate solution (VWR Prolabo, cat. no. 24254.293) allowed to react in a boiling water bath for 50 min and then cooled rapidly on ice. Absorbance was read at 550 nm in a Bio-Rad model 680 microplate reader and concentration of condensed tannins (TANN, mg g^−1^) estimated using as standard, purified condensed tannins of the quebracho tree (*Schinopsis balansae* Engl.; Droguería Moderna, Pontevedra, Spain).

### Data analysis

Univariate statistical analysis of all the variables was performed using linear mixed models with two between-subjects fixed factors, the bending treatment (control and bent) and the provenance group (straight and crooked types), and different within-subject fixed factors depending on the variable considered. For A01, DIFA01, A12, DIFA12, and LENG, the models included one within-subjects factor, the time after releasing, and an unstructured covariance matrix for each treatment was used to model the random errors. For S1MF, LMF, S2MF, and S1LMF, assessed only once at the end of the experiment, the models did not include any within-subjects, but different residual variances were considered for each of the four treatment×provenance group combinations. In the case of pCW, the models included the three different sections as a within-subjects factor assuming a compound symmetry covariance matrix across sections and heterogeneous residual variances across the four treatment levels. Finally, the models used for analysing WDENS, STARCH, SUGAR, TANN, and PHEN had the orientation as a within-subjects factor with an unstructured covariance matrix across orientations and heterogeneous residual variances across the two provenance groups. The tree was the experimental unit in all the models, so it was not included as a random factor. The variance between trees was the residual variance in the models. In all cases, the models included the provenance (nested within the provenance group) as a random factor (see Supplementary Table S2 for a detailed description of random and fixed factors in the models). Individual contrasts and tests of effect slices were performed for all the comparisons in the models.

Normality of the Studentized residuals in the linear mixed models was checked using a Kolmogorov–Smirnov test. All the *P*-values were above 0.005 and half the time they were above 0.05. The deviations from normality were not relevant, as seen in the probability plots. The degree of fit to the bisector in the normal probability plot was measured using Lin’s concordance correlation coefficient, and all values were above 0.95. The adequacy of the variance structures was checked with the null model likelihood ratio test. The *P*-values were mostly below 0.005, which proved the need to use mixed models.

Principal components analysis, separated for control and bent plants, was performed to analyse the relations between the following variables: DIFA01, pCW, WDENS, LENG_45_, TDW, S1_L_MF, STARCH, SUGAR, TANN, and PHEN. The differences between the Pearson correlation coefficients for control and bent plants were checked by using Cohen’s *q*-test for two independent samples (Cohen, 1977). The procedures MIXED and PRINCOMP of SAS 9.4 (SAS Institute Inc., 2011) were used for statistical analyses.

## Results

### Straightening kinetics and elongation

**Fig. 3.**
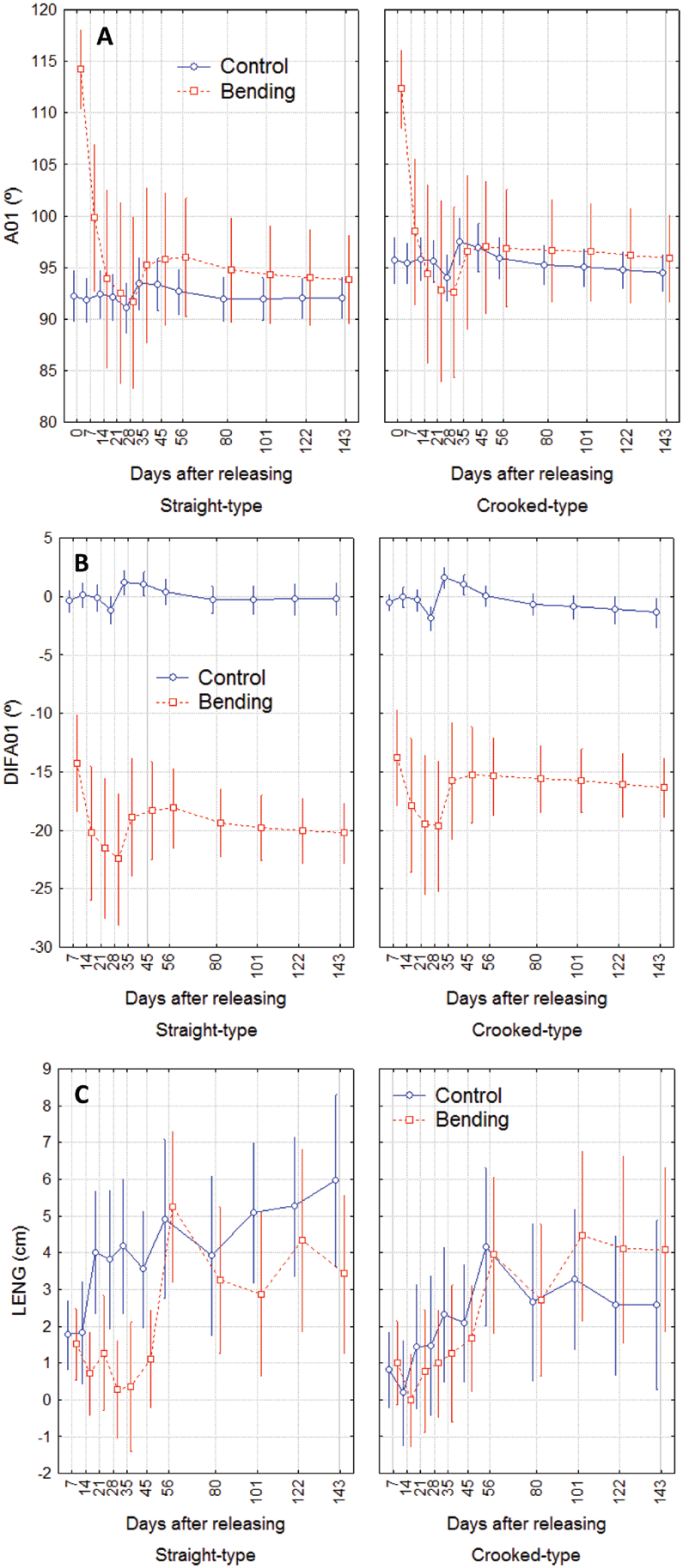
Evolution of plant straightening kinetics and elongation after releasing from bending at each measuring date. (A) Basal angle (A01). (B) Difference of angle from maximum bending (DIFA01). (C) Stem elongation (LENG). Bars represent 95% confidence intervals for the mean.

In the control treatment, angle A01 averaged between 93º and 94º throughout the experiment, with slightly but significantly lower values for crooked-type plants (i.e. less vertical) compared with straight-type ones at all measurement dates (Fig. 3A). In the bending treatment, the mean A01 before and after bending was 94° and 125°, respectively. Just before releasing the plants at the end of the bending period, the mean A01 was 121º and immediately after release, mean A01 dropped to 114º. Straightening was very fast after release: A01 reached 99.5º during the first week and 94.5º during the second week. From that moment on, A01 fluctuated slightly until the end of the growth period (end of October) with an average of 95º. In the bending treatment, there were no significant differences in A01 between straight-type and crooked-type plants at any date, indicating a similar straightening reaction after releasing (Fig. 3A). DIFA01 was not significantly different between straight- and crooked-type plants up to 100 d after release, but after that date the straight-type plants continued approaching verticality and attained 4º closer to the vertical than the crooked-type plants (*F*=4.39; *P*=0.0365) (Fig. 3B). In the apical segment, the response (A12 and DIFA12) consisted of an initial sagging phase, a subsequent decrease to an average of 63º for a week and then a period of recovery of verticality up to 90º during 1 month. No significant differences were observed between provenance groups (types) and treatments at any date (Supplementary Fig. S2).

Stem elongation during the first 45 d after release was significantly lower in bent plants compared with the control ones only in the straight-type, while the crooked-type bent plants maintained the same elongation pattern as the control at all dates until day 45 (Fig. 3C). After this period, the straight-type bent plants restored their elongation, reaching the same length as the control plants in the next 11 d. For both plant types, no significant differences between treatments were observed from day 45 until the end of the experiment.

### Biomass allocation

**Fig. 4.**
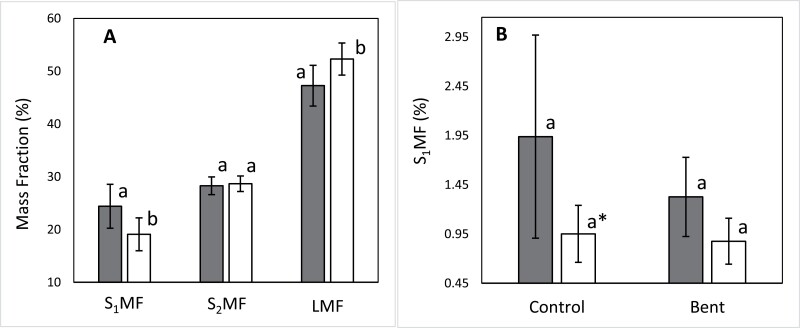
(A) Mass fractions of main stem, branch stems, and leaves (S1MF, S2MF, and LMF respectively, both treatments averaged). (B) Comparison of main stem mass fraction (S1MF) per treatment (control and bent) and provenance group (straight-type and crooked type). Straight-type: dark bars; crooked-type: white bars. Different letters indicate significant (*P*<0.05) differences between types, except ∗*P*<0.1. Different letters indicate significant (*P*<0.05) differences between types, both treatments pooled (A) and per treatment (B). Bars represent 95% confidence intervals for the mean.

Straight-type plants showed higher allocation to the main stem (*F*=4.26; *P*=0.0450) and less allocation to leaves (*F*=4.29; *P*=0.0442) than crooked-type ones, with no significant differences between the two plant types in the mass fraction corresponding to branch stems (Fig. 4A). Treatment and treatment×type interaction were non-significant for any of these fractions.

Biomass allocation to the leader shoot (developed during the year of the experiment) was higher in the straight-type plants compared with the crooked-type ones in both treatments, although differences were only marginally significant (*P*<0.10) (Fig. 4B).

### Wood density and compression wood

Compression wood was found mainly in bent plants at the south side of the sections, as a consequence of a gravitropic reaction to the bending while in some of the upper sections of the bent plants, compression wood was also found in the north side (autotropic reaction).

**Fig. 5.**
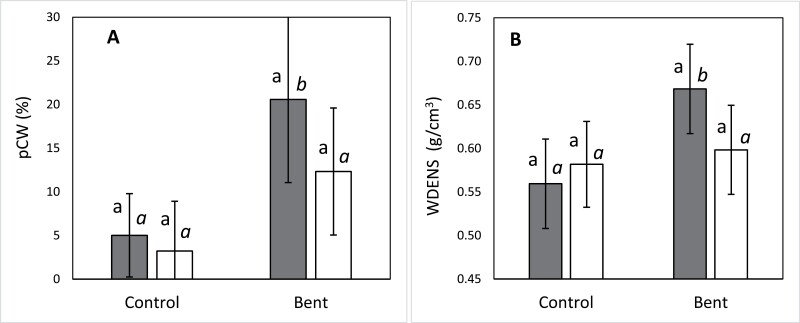
Percentage of compression wood in the middle section (A) and wood density at the basal section (B) of the ring developed during the year of the experiment per treatment (control and bent) and provenance group (straight-type: dark bars; crooked-type: white bars). Different roman letters indicate significant (*P*<0.05) differences between types within treatments, while different italic letters indicate significant (*P*<0.05) differences between treatments for each type. Different roman letters indicate significant (*P*<0.05) differences between types within treatments, while different italic letters indicate significant (*P*<0.05) differences between treatments for each type. Bars represent 95% confidence intervals for the mean.

The percentage of compression wood in the ring developed during the experiment (pCW) was significantly affected by both the treatment and the section (i.e. the height of the trunk where samples were taken) (*P*=0.00 and *P*<0.001, respectively; Supplementary Fig. S3). When looking at each section individually, significant differences between treatments only occurred in the middle one, with bent plants showing greater percentage of compression wood than control plants (Fig. 5A). Straight-type plants showed higher pCW in the bending treatment compared with the control (*F*=8.33, *P*=0.005), while in the crooked-type plants the treatment did not induce significant changes (*F*=3.79, *P*=0.054).

The bending treatment induced an increase of wood density in the annual ring formed during the experiment compared with control plants at the basal section (*F*=6.17, *P*=0.016), but the effect was markedly different depending on the provenance group: only the straight-type plants responded to the bending treatment by increasing wood density 19.5% (*P*=0.004), while the bending treatment had no significant effect in the crooked-type ones (Fig. 5B).

### Non-structural carbohydrates and secondary C metabolites

**Fig. 6.**
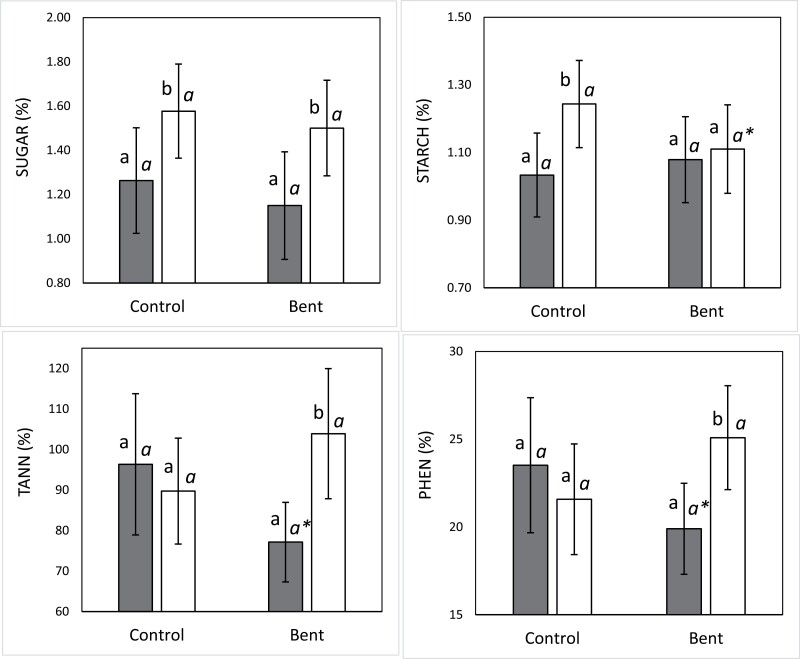
Comparison of non-structural carbohydrates and secondary C metabolites per treatment (control and bent) and provenance group (straight-type: dark bars, crooked-type: clear bars). (A) Sugar, (B) starch, (C) tannins, and (D) phenolics. Different roman letters indicate significant (*P*<0.05) differences between types within treatments, while different italic letters indicate significant (*P*<0.05) differences between treatments for each type. Bars represent 95% confidence intervals for the mean.

The concentration of soluble sugars was significantly higher in crooked- than in straight-type plants (*F*=5.69; *P*=0.021), regardless of the treatment. Crooked plants showed 27% higher sugar concentration than straight plants overall. The starch concentration was also significantly higher in crooked- than in straight-type plants, but only in the control treatment (*P*=0.022). In this case, crooked plants showed 20% higher sugar concentration than straight plants. In the bending treatment, the starch concentration decreased marginally compared with the control in the crooked-type plants (*P*=0.089), but not in the straight-type ones (*P*=0.506) (Fig. 6A, B).

In the control treatment both types of plants had similar levels of condensed tannins and total polyphenolics, while in the bending treatment these traits were significantly higher in crooked- than in straight-type plants (35% higher, *P*=0.006 for tannins and 26%, *P*=0.011 for polyphenolics). Moreover, the reaction to the bending treatment caused a marginally significant decrease both in tannins and phenolics in the straight-type plants (*P*=0.055 and *P*=0.099, respectively), while no significant change occurred in the crooked-type ones (*P*=0.175 and *P*=0.109) (Fig. 6C, D).

### Multivariate analysis

**Fig. 7.**
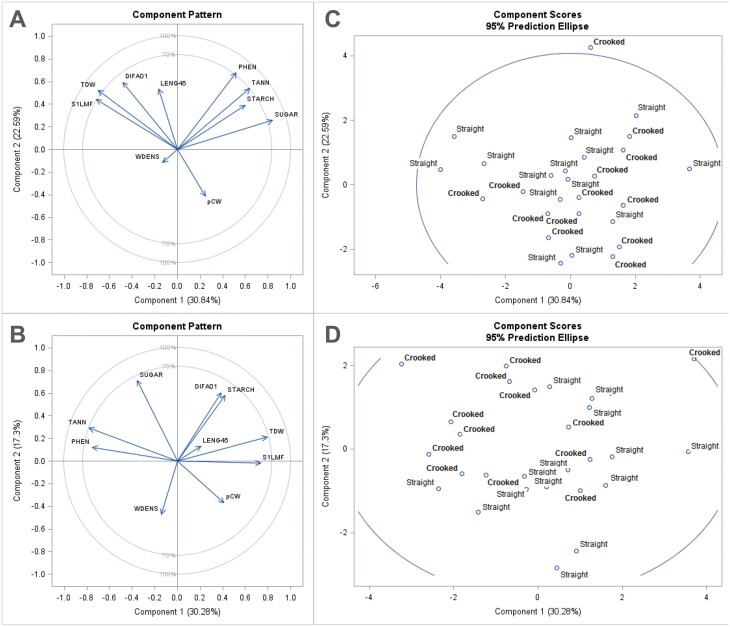
Plots of component pattern and scores corresponding to the first two principal components, per treatment. (A, C) Control treatment; (B, D) bending treatment. In (C, D) each point indicates an individual plant, identified by its provenance group (straight or crooked).

The principal component analysis on the ten study variables (DIFA01, pCW, WDENS, LENG_45_ TDW, S1_L_MF, STARCH, SUGAR, TANN, and PHEN) showed four components with eigenvalues >1, which represented 80.0% and 76.0% of the variation for control and bending treatments, respectively (Fig. 7A, B; Supplementary Table S3). In the control treatment, 53.4 % of the variation was captured by the first two components, being S1_L_MF, SUGAR, and PHEN, the variables with higher contribution. In the bending treatment the first two components captured 47.6% of the variation, and the variables that contributed most were S1_L_MF, TDW, TANN, PHEN, DIFA01, SUGAR, and STARCH (Supplementary Table S3).

Plots with the first two principal components showed a low segregation between crooked- and straight-type plants in the control treatment, but a much higher segregation in the bending treatment, with straight-type plants grouped right of the diagonal (higher pCW and WDENS and lower SUGAR, TANN, and PHEN), while crooked-type plants grouped left of the diagonal correlated to the opposite traits (Fig. 7C and C). Plots with other principal components did not show any distinct pattern of segregation between types.

**Fig. 8.**
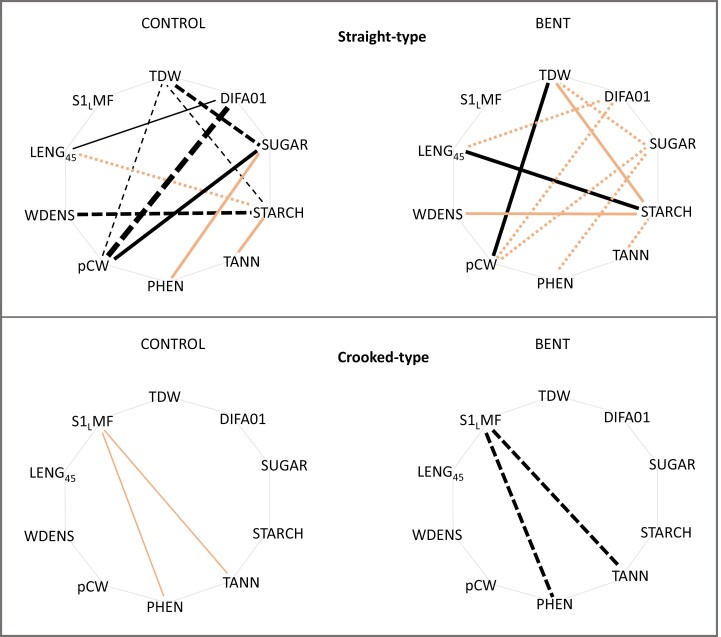
Change of pairwise correlations between treatments for straight- and crooked-type plants. Line thickness is proportional to the magnitude of the correlation coefficient. Full lines represent positive correlations and dotted lines represent negative correlations. Black lines indicate significant correlations (*P*<0.05), orange lines indicate non-significant correlations, but with a reciprocal correlation in the other treatment which Cohen’s *q*-statistic indicated as significantly different.

The comparison of pairwise correlations between control and bent plants using Cohen’s *q*-test showed that 10 pairwise correlations changed significantly in the straight-type plants, while only two correlations changed in the crooked-type plants (Fig. 8; Supplementary Table S4).

## Discussion

In this study, we took advantage of the well-documented intraspecific differentiation in stem form in a model conifer (*Pinus pinaster*) to perform a manipulative experiment of stem postural control including a wide range of phenotypic traits. While previous studies characterized the responses to bending in 2-year-old seedlings of three provenances of the species (Sierra-de-Grado *et al.*, 2008), the present work considerably broadens the scope to a more advanced ontogenetic stage while covering a wider range of provenances.

Crooked-type plants were less straight than straight-type plants of the same age (9 years) under the same experimental field environment and without any intervention. This confirms the already known intraspecific variation of the trunk shape among maritime pine provenances, highly consistent across environments (Alía *et al.*, 1995; Sierra de Grado *et al.*, 1999). Moreover, after when experimentally bent, straight-type plants recovered verticality faster and at a higher degree compared with crooked-type plants, thereby confirming our second hypothesis and, also, in agreement with previous results using younger seedlings of this species (Sierra-de-Grado *et al.*, 2008). The main differences in the response to bending and releasing between the two provenance groups relied on the secondary growth of the stem (represented by A01 and DIFA01), rather than on the plasticity of the apical part with primary growth.

Family and individual differences have also been found using similar experimental approaches in *Pinus radiata* (Lachenbruch *et al.*, 2010; Apiolaza *et al.*, 2011) and *P. pinaster* (Sierra-de-Grado *et al.*, 1997). This intraspecific variation has been also related to certain thresholds of mechanical stimulus (degree of inclination) that shift the speed of recovering verticality (Lachenbruch *et al.*, 2010).

Both straight and crooked types showed similar phenological patterns of shoot elongation in the control treatment. In contrast, after having been bent, both provenance groups responded differently in stem elongation. While straight-type plants delayed shoot elongation (consistent with previous results in different woody and non-woody plant species; Meng *et al.*, 2006; Martínez-Zurimendi *et al.*, 2009; Coutand, 2010), crooked-type plants did not show a significant early decrease in elongation as a response to bending. Interestingly, both plant types attained similar total elongation at the end of the experiment, indicating an efficient recovery of growth despite the initial delay in straight-type plants.

Confirming our second hypotheses, and in agreement with the literature (Schwilk and Ackerly, 2001; Garrido *et al.*, 2015), straight-type plants dedicated comparatively more biomass to the main stem, while crooked-type plants dedicated more resources to leaves, hence the crooked-type plants ‘ignored’ to some extent the function of mechanical support. This is in line with a divergence between life history strategies observed in comparative studies among species: a long-lived strategy characterized by straight stems and high allocation to the main stem, and a short-lived strategy with the opposite trend (Van Gelder *et al.*, 2006; Poorter *et al.*, 2010). The bending treatment had little influence on biomass allocation, which was somewhat expected for 9-year old plants submitted to the manipulative experiment only during their last year. Moreover, biomass allocation to the leader shoot (formed during the experiment) was always higher in straight-type plants, with no clear effect of the bending treatment, in line with the results previously mentioned. Even when root biomass was not assessed in our experiment, in a previous work with tilted containerized seedlings of *P. pinaster*, allocation to roots (about 30% of total biomass) did not vary significantly between the two provenance groups, and the main difference between straight and crooked provenances was biomass allocation to the main stem (Garrido *et al* 2015).

The formation of compression wood in response to bending in the ring formed during the experiment was clearly different between the two provenance groups: straight-type plants formed a higher percentage of compression wood in response to bending, especially in the medium section of the trunk where the tension was applied. By contrast, crooked-type plants did not show changes in this trait, which is, however, central to the mechanism of stem postural control in woody plants. Reaction wood introduces heterogeneity in wood properties, which in turn generates asymmetry in mechanical stress (maturation strains) around the circumference of the tree and works as the main stem straightening ‘motor’ (Wilson and Archer, 1979; Moulia and Fournier, 2009). The different performance in compression wood formation between provenance groups in our experiment had a parallel response in the wood density of the basal section of the trunk, i.e. denser wood of the last growth ring in response to bending in the straight- but not in the crooked-type plants. Moreover, we found no differences in wood density between the two plant types (i.e. provenance groups) in the control treatment, even when a higher density in straight-type plants could have been expected, considering the comparisons of life-history strategies between species (Van Gelder *et al.*, 2006; Poorter *et al.*, 2010).

In addition to mechanical properties, the presence of compression wood can also affect other xylem functions such as water transport, defence against pathogens and carbohydrate storage (Fournier *et al.*, 2014). Compression wood formation involves multiple modifications in gene expression and regulation factors that lead to changes in lignification and polysaccharide metabolism (Plomion *et al.*, 2000; Yeh *et al.*, 2006; Fagerstedt *et al.*, 2014; Donaldson and Singh, 2016). Other works also report a higher concentration of monosaccharides (galactans, pentoses, etc.) in the reaction wood (Nanayakkara *et al.*, 2005; Mast *et al.*, 2009; Chavan *et al.*, 2015), which in conifers has been associated with a higher concentration of lignin (Diaz-Vaz *et al.*, 2009, Hiraide *et al.*, 2016).

While starch mainly represents the long-term reservoir of the non-structural carbon pool of plants, sugars are a far more movable and labile, ready-to-use source of energy (Martínez-Vilalta *et al.*, 2016). Therefore, considering that both types of carbohydrates were measured at the end of the experiment, we initially expected differences in starch (but not in sugar) concentration associated with the bending treatment. The results were consistent with this expectation in the case of crooked-type plants, but not for straight-type plants. Only the crooked-type plants showed a decrease in starch concentration (denoting consumption of stored reserves), while no effect of the bending treatment on starch dynamics was observed in straight-type plants, as also observed in an artificial defoliation experiment of young *P. pinaster* trees of about the same age as this experiment (Puri *et al.*, 2015). This raises the question of the destination of the energy obtained from starch in crooked-type plants submitted to bending, since these plants did not produce denser wood, more compression wood or more primary or secondary growth. A possible, yet unexpected, explanation could be related to the different tannin and polyphenol contents in crooked-type plants following bending compared with straight type. These secondary metabolites are generally related to defence responses to herbivory and other biotic stressors, including mechanical wounding (Moreira *et al.* 2013; Jacobo-Velázquez *et al.*, 2015). Although we are not aware of previous studies linking plastic responses involving these compounds as a reaction to bending, results presented here suggest that they could play some role in this process. Both types of compound tended to decrease in straight-type plants but to increase in crooked-type plants in response to bending (both responses were, however, only marginally significant). The decrease of these compounds in straight-type plants, in which bending did not diminish stored reserves but induced notable changes in wood density and compression wood, could reflect a mobilization of these compounds to produce such a response. Alternatively, the increase of both types of compounds in crooked-type plants in response to bending could be related to a less specific response to mechanical stress, including the consumption of reserve carbohydrates.

In agreement with our second hypothesis, the principal component analysis considering all variables analysed confirmed a neat differentiation in the response to bending between straight- and crooked-type plants. In particular, the bending treatment caused a marked segregation between both provenance groups, while this did not occur in the control treatment. The more influential traits causing such segregation were highly coherent with the univariate analyses: after bending, straight-type plants had a higher percentage of compression wood and wood density and lower tannin and polyphenol contents, while crooked-type plants showed the opposite trend. This differentiation in the combined response of multiple traits can be evaluated under the theoretical framework of phenotypic integration since different phenotypic plasticity of each individual trait leads to a different constellation of correlated traits between treatments (Pigliucci and Marlow, 2001; Santini *et al.*, 2020). This is quite evident looking at Fig. 7—derived from Cohen’s *q*-coefficient for pairwise correlation comparison—where straight-type plants displayed far more significant changes of correlations between the control and bending treatments (10 trait pairs) compared with crooked-type plants (two pairs).

Altogether, results presented here indicate different responses to mechanical stimuli between provenance groups with a different usage of the resources linked to the intraspecific, genetically based variation of our study species. The straight-type provenances displayed a more efficient resource usage, transitorily delaying shoot elongation, developing compression wood while reducing resource allocation to defence and, notably, maintaining the levels of stored non-structural carbohydrates, indicative of an adaptive multi-trait plasticity in which postural control plays an important role. In contrast, the lack of these expected responses in crooked-type provenances could suggest a lower fitness value of straight stems in this provenance group. Further research would be needed to unveil the environmental conditions that favours this divergence, putatively through directional selection as postulated for other traits (Correia *et al.*, 2008; Santos-del-Blanco *et al.*, 2012; de la Mata *et al.*, 2012).

Our first hypothesis was that the straightening process is costly in terms of growth or other currencies, but our experiment was scarcely conclusive on this point, showing only minor or transient costs in straight-type plants. We have measured a limited, albeit representative, number of morphological and physiological traits, either directly connected to the mechanical response or usually used as indicators of defence and maintenance functions. However, many other traits could be also involved in the straightening process. For example, there is strong evidence that bark plays a key role in stem straightening in broadleaved trees and that this contribution varies among species, ontogenetic stage, bark relative thickness, and anatomical features (Niklas, 1999; Poorter *et al.*, 2014; Lehnebach *et al.*, 2020; Clair *et al* 2019). Therefore, the role of bark in the straightening process should be investigated in longer term, future studies in *P. pinaster*. Moreover, monitoring traits related to photosynthetic activity and water balance could also increase our understanding of the complex plasticity of the integrated phenotype related to mechanical responses (Taneda and Tateno, 2004; Niklas *et al.*, 2006; Martone *et al.*, 2010). Finally, given the destructive nature of our experiment, it was limited in time, and hence we still do not know if there were longer term effects on alternative key fitness traits, namely reproduction and survival, which would increase our understanding of the ecological and evolutionary implications of stem postural control.

## Supplementary data

The following supplementary data are available at *JXB online*.

Fig. S1. Performance of a bent tree in the experiment.

Fig. S2. Evolution of the apical part of the stem after releasing from bending at each measuring date.

Fig. S3. Comparison of the percentage of compression wood in the ring developed during the year of the experiment at three stem heights.

Table S1. Sample description: plants per provenance grouped into straight- and crooked-types (based on their stem growth habit in common gardens) and treatment, control and bent.

Table S2. Random and fixed factors included in the linear mixed models for each variable.

Table S3. Principal component analysis.

Table S4. Cohen’s *q*-coefficients.

erab535_suppl_Supplementary_Materials

## Data Availability

The data supporting the findings of this study are available from the corresponding author (RSG), upon request.

## Abbreviations

A01: angle with respect to the horizontal of the basal part of the stem (º)
A12: angle with respect to the horizontal of the apical part of the stem (º)
DIFA0: difference of angle A01 at each date with respect to A01 just after releasing (º)
DIFA12: difference of angle A12 at each date with respect to A12 just after releasing (º)
LENG: length of the apical segment (cm)
LMF: leaf mass fraction (%)
pCW: percentage of compression wood area over the total yearly ring area (%)
PHEN: total phenolics concentration (mg g^−1^)
S1MF: main stem (leader shoot included) mass fraction (%)
S1LMF: leader shoot mass fraction (%)
S2MF: branch stem mass fraction (%)
STARCH: starch (%)
SUGAR: soluble sugar concentration (%)
TANN: condensed tannins concentration (mg g^−1^)
TDW: total dry weight (sum of main stem (leader shoot included), leaf, and branches dry weight) (g)
WDENS: wood density of the yearly ring (g cm^−3^)
