# Supplementary material for: Straightening the crooked: intraspecific divergence of stem posture control and associated trade-offs in a model conifer
Source: J Exp Bot. 2021 Dec 5;73(4):1222–35. doi: 10.1093/jxb/erab535 (PMC8866635; doi:10.1093/jxb/erab535)
Supplement: erab535_suppl_Supplementary_Materials [file erab535_suppl_supplementary_materials.pdf]

Supplementary data

Table S1. Sample description: plants per provenance grouped into straight- and crooked-types (based on their stem growth habit in common gardens) and treatment, control and bent.

Type	Provenance	No. control plants	No. bent plants	Total
Straight	Burgos-Soria	4	5	9
	Gredos	4	4	8
	Leiría	2	3	5
	Tamjout	4	3	7
Crooked	Almijara	4	4	8
	Castillian Plateau	5	5	10
	Oña	6	6	12
Total		29	30	59

Table S2. Random and fixed factors included in the linear mixed models for each variable

	Random	Fixed factors				
	Factor	Between-subjects			Within-subjects	
	Provenance	Type	Treatment	Date	Orientation	Section
A01, DIFA01, A12, DIFA12, LENG	X	X	X	X		
S1MF, S2MF, LMF, S1LMF, TANN, PHEN	X	X	X			
pCW	X	X	X			X
WDENS, STARCH, SUGAR	X	X	X		X	

Table S3. Principal component analysis (in bold significant components within auto-vectors, in brackets % of explained variance for each autovector). Principal Components with eigenvalues > 1 are shown in Control and Bending treatments.

CONTROL	Autovectors			
	PC1 (30,84%)	PC2 (22,59%)	PC3 (14,67%)	PC4 (11,86%)
S1LMF	-0.40876	0.29400	-0.40876	0.03557
TDW	-0.39847	0.34844	-0.01127	-0.00152
DIFA01	-0.27562	0.39522	-0.19746	-0.17491
pCW	0.13964	-0.27395	0.40759	-0.19227
SUGAR	0.47834	0.17032	-0.11777	0.31261
STARCH	0.34044	0.26134	-0.29726	0.48355
TANN	0.36349	0.35766	0.19424	-0.41988
PHEN	0.29598	0.45075	0.24847	-0.35002
LENG 45	-0.09346	0.35605	0.41487	0.44649
WDENS	-0.07419	-0.07573	0.64767	0.31852

BENT	Autovectors			
	PC1 (30,28%)	PC2 (17,30%)	PC3 (15,70%)	PC4 (12,69%)
S1LMF	0.42204	-0.01474	-0.17800	0.38059
TDW	0.45521	0.16241	-0.08480	0.34294
DIFA01	0.22213	0.46081	-0.23318	0.16185
pCW	0.23608	-0.28015	0.41130	0.27845
SUGAR	-0.20534	0.54153	0.27604	-0.20625
STARCH	0.24270	0.44011	0.47528	-0.16593
TANN	-0.44894	0.22277	0.01407	0.45843
PHEN	-0.43168	0.09352	-0.00504	0.53983
LENG45	0.11859	0.10084	0.44798	0.22623
WDENS	-0.08100	-0.35649	0.48434	0.10481

Table S4. Cohen-q test. Only significant pairwise comparisons are shown in the table (LC and UC lower and upper limits of the 95% and 90% confidence intervals). Column Type indicates if the comparison is significant for straight- or crooked-type plants.

VAR1	VAR2	RControl	NControl	RBent	NBent	N	qCohen	LC95	UC95	LC90	UC90	Type
TDW	pCW	-0,4808	14	0,5520	14	28	-0,4974	-0,8894	-0,1054	-0,8264	-0,1684	Straight
STARCH	WDENS	-0,6180	14	0,2126	14	28	-0,4072	-0,7992	-0,0152	-0,7362	-0,0783	Straight
TDW	STARCH	-0,4932	14	0,3636	14	28	-0,4001	-0,7921	-0,0081	-0,7291	-0,0711	Straight
STARCH	LENG45	-0,2643	14	0,5682	14	28	-0,3977	-0,7897	-0,0057	-0,7267	-0,0687	Straight
DIFA01	pCW	-0,8170	14	-0,2473	14	28	-0,3887	-0,7807	0,0033	-0,7177	-0,0597	Straight
TDW	SUGAR	-0,6883	14	-0,0468	14	28	-0,3465	-0,7385	0,0455	-0,6755	-0,0175	Straight
SUGAR	PHENN	0,4426	14	-0,2950	14	28	0,3385	-0,0535	0,7305	0,0095	0,6675	Straight
pCW	SUGAR	0,5866	14	-0,1144	14	28	0,3419	-0,0501	0,7339	0,0130	0,6709	Straight
S1LMF	TANN	0,0651	14	-0,6578	14	28	0,3709	-0,0211	0,7629	0,0420	0,6999	Crooked
STARCH	TANN	0,3620	14	-0,4541	14	28	0,3774	-0,0146	0,7694	0,0484	0,7064	Straight
DIFA01	LENG45	0,4591	14	-0,4135	15	29	0,4065	0,0221	0,7909	0,0839	0,7291	Straight
S1LMF	PHEN	0,2291	14	-0,6132	14	28	0,4114	0,0195	0,8034	0,0825	0,7404	Crooked

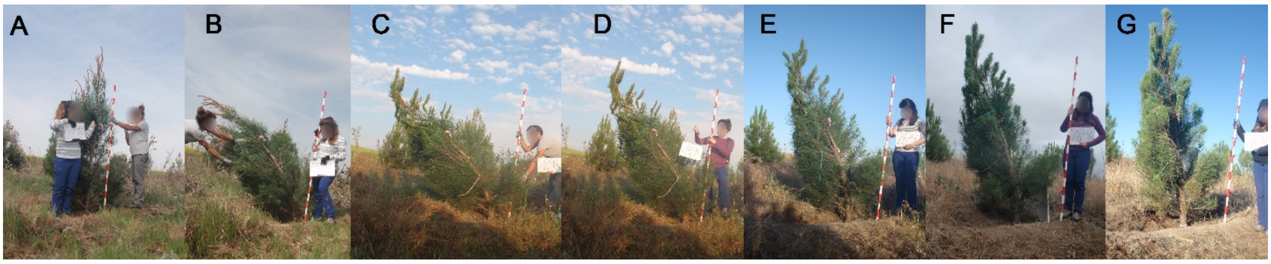

Figure S1. Performance of a bent tree on the experiment. A and B, same date before and after bending; C and D one month later, before and after release; E, F and G 14, 35 and 143 days after release, respectively.

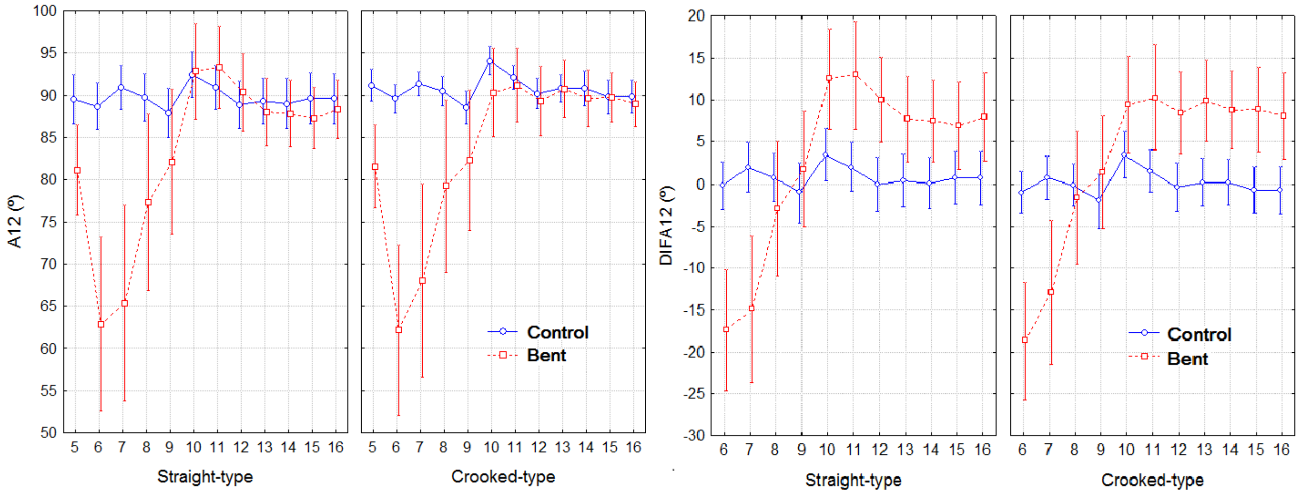

Figure S2. Evolution of the apical part of the stem after releasing from bending at each measuring date. A) Angle respect to the horizontal of the apical part of the stem (A12), B) Difference of angle with respect to A12 just after releasing (DIFA12). Bars represent 95% confidence intervals for the average.

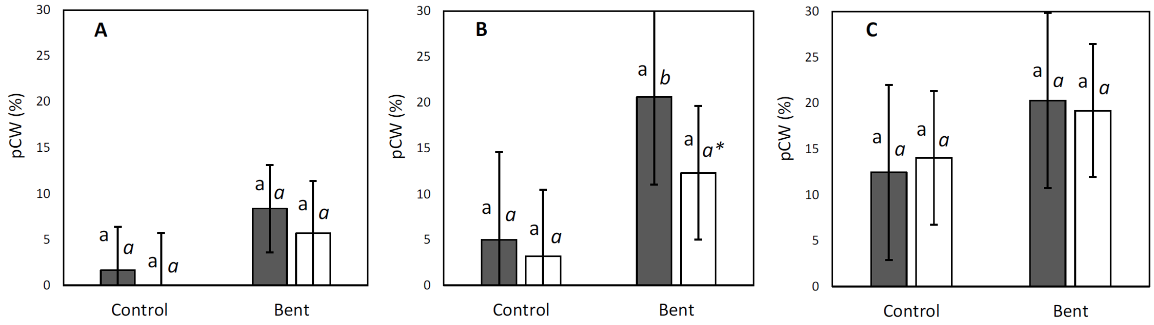

Figure S3. Comparison of the percentage of compression wood in the ring developed during the year of the experiment at three stem heights: A) distal (X, under the 1-year-old branches whorl), B) middle (Y, under the 4-years old branches whorl) and C) basal (Z, ground level) per provenance group (straight-type: dark bars; crooked-type: white bars). Different roman letters indicate significant ($p<0.05$) differences between types within treatments, while different italic letters indicate significant ($p<0.05$) differences between treatments for each type. Bars represent 95% confidence intervals for the average.
